# Spatial and temporal variations of net ecosystem productivity in Xinjiang Autonomous Region, China based on remote sensing

**DOI:** 10.3389/fpls.2023.1146388

**Published:** 2023-02-14

**Authors:** Xiangjun Lu, Yang Chen, Yuyin Sun, Yongming Xu, Yan Xin, Yaping Mo

**Affiliations:** ^1^ The First Ecological and Environment Monitoring Station of Xinjiang Production and Construction Corps, Urumchi, China; ^2^ School of Remote Sensing and Geomatics Engineering, Nanjing University of Information Science & Technology, Nanjing, China; ^3^ Institute of Geographic Sciences and Natural Resources Research, Chinese Academy of Sciences, Beijing, China

**Keywords:** NEP, spatio-temporal variations, remote sensing, Xinjiang, CASA

## Abstract

Net ecosystem productivity (NEP), which plays a key role in the carbon cycle, is an important indicator of the ecosystem's carbon budget. In this paper, the spatial and temporal variations of NEP over Xinjiang Autonomous Region, China from 2001 to 2020 were studied based on remote sensing and climate re-analysis data. The modified Carnegie Ames Stanford Approach (CASA) model was employed to estimate net primary productivity (NPP), and the soil heterotrophic respiration model was used to calculate soil heterotrophic respiration. Then NEP was obtained by calculating the difference between NPP and heterotrophic respiration. The annual mean NEP of the study area was high in the east and low in the west, high in the north and low in the south. The 20-year mean vegetation NEP of the study area is 128.54 gC·m^-2^, indicating that the study area is a carbon sink on the whole. From 2001 to 2020, the annual mean vegetation NEP ranged between 93.12 and 158.05 gC·m^-2^, and exhibited an increasing trend in general. 71.46% of the vegetation area showed increasing trends of NEP. NEP exhibited a positive relationship with precipitation and a negative relationship with air temperature, and the correlation with air temperature was more significant. The work reveals the spatio-temporal dynamics of NEP in Xinjiang Autonomous Region and can provide a valuable reference for assessing regional carbon sequestration capacity.

## 1 Introduction

Since the industrial revolution, the concentration of carbon dioxide in the atmosphere has dramatically increased with the development of technology and the widespread use of fossil fuels. In 1705, the global atmospheric CO_2_ concentration is 278 ppm, but in 2022, it has reached 417 ppm, an increase of about 50% (Met Office, 2022; NOAA, 2022). The increase in atmospheric CO_2_ concentration has caused a series of climate and environmental problems, including global warming, extreme weather events, sea level rising, and glacier retreat, which have important impacts on human survival and development (Zhao and Luo, 1998; Hoegh and Bruno, 2010; IPCC, 2014; Baker et al., 2018; Luo and Keenan, 2022; Xue and Qin, 2022). Carbon cycle research has attached strong interest from governments and scientists. Terrestrial ecosystem is an important part of the global carbon cycle and a key indicator of regional environment and human activities (Schimel et al., 2015; Friend et al., 2014). Net ecosystem productivity (NEP), which is the difference between net primary productivity (NPP) and soil heterotrophic respiration (Rh), can effectively indicate carbon absorption and emission capacity, and is generally used as an indicator of carbon source or carbon sink (Keenan et al., 2016; Li et al., 2020). A positive NEP means that the carbon absorbed by the ecosystem is more than the carbon emitted, which is a carbon sink. A negative NEP indicates that the carbon absorbed by the ecosystem is less than the carbon emitted, which is a carbon source. Accurate knowledge of NEP is critically important for assessing regional carbon sequestration capacity and developing strategies to stabilize the CO_2_ emissions.

Traditional investigation methods of NPP include the biomass survey method, the direct harvest method, Eddy Covariance method, chlorophyll estimation method, and the raw material consumption measurement method. Most of these methods are time-consuming and can only provide NPP estimations at small scales. Recently, satellite remote sensing data are employed to map NPP at large scales, and several methods were developed for estimating NPP from remote sensing data, including light energy utilization models, physiological and ecological process models, and climate productivity models (Goetz et al., 1999; Parton et al., 1993; Veroustraete et al., 1994). Among these methods, the Carnegie-Ames-Stanford Approach (CASA) model, which is a simple light energy utilization model that uses remotely sensed normalized difference vegetation index (NDVI) and meteorological data as input variables, has been widely used. Many studies have been carried out on the spatial and temporal variations of NPP based on remote sensing datasets (Bradford et al., 2005; Chirici et al., 2007; Pachavo and Murwira, 2014; Ji et al., 2021; Xue et al., 2021; Mngadi et al., 2022). NPP can well quantify the carbon absorption by plants; while NEP can depict both the carbon absorption by plants and carbon release by soils. Compared with NPP, NEP can better depict regional carbon sequestration capacity. However, there are quite fewer studies investigating NEP from remote sensing at large scales. Yamaji et al. (2008) combined Moderate Resolution Imaging Spectroradiometer (MODIS) data and flux-based observations to obtain NEP maps of deciduous forests in Japan for the years 2002-2003 using a scaling-up technology. Nayak et al. (2015) analyzed the spatial and temporal variations of NEP over India during 1981–2006 using the CASA model and regional database and also investigated its relationship with climatic parameters. He et al. (2018) used the CASA model and a soil microbial respiration model to estimate NEP in the Yellow River Basin during 1982-2015 and analyzed its spatial response under diurnal asymmetric warming. Zhao et al. (2019) used an improved individual-based forest ecosystem carbon budget model and Advanced Very High Resolution Radiometer (AVHRR) remote sensing data to estimate the NEP of global forest ecosystems from 1982 to 2011, and then investigated the impacts of climate change on the NEP of different forest types. Guo et al. (2021) applied the CASA model and a heterotrophic respiration model to map NEP in the Hindu Kush Himalayan region and studied the temporal and spatial changes of NEP magnitude from 2001 to 2018. Zhang J. et al. (2021) combined the CASA and empirical models to map the NEP in Central Asia during 2001-2019 and evaluated the impact of drought on the carbon source and sink.

Xinjiang Autonomous Region is located in an arid and semi-arid climate zone in northwestern China. It is one of the most sensitive regions to climate change, which is characterized by relatively low ecosystem productivity and weak system regulation capacity. Quantitative investigating the ecosystem NEP in this region and the response to climate is of great significance. This paper aims to integrate satellite remote sensing data and meteorological reanalysis data to study the spatial and temporal variations of net ecosystem productivity in the region in the past 20 years, which is of great significance for the understanding of changes in carbon balances and expenditures in Xinjiang, and can provide a reference for the regulation of carbon cycle and ecological protection strategy in the region.

## 2 Study area and data

### 2.1 Study area

**Figure 1:**
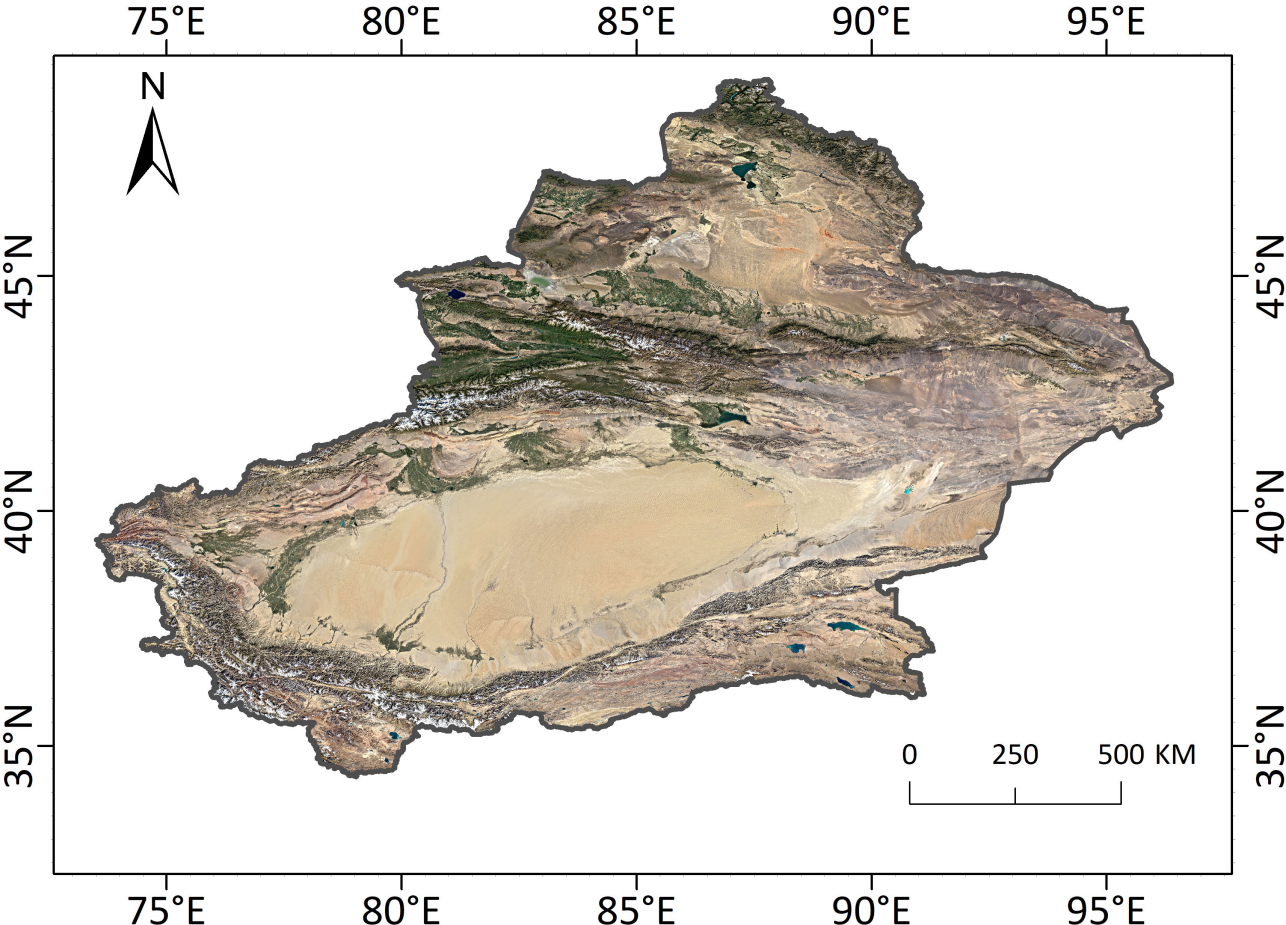
Study area.

Xinjiang Uygur Autonomous Region is located in the northwest of China (**Figure 1**). It ranges from 73°20' to 96°25' E and 34°15' to 49°10' N, with a total area of about 1.66 million km^2^. The region has a complex and diverse topography, with the Altai, Tianshan, and Kunlun Mountains distributed from north to south. The Junggar and Tarim basins are located on the north and south sides of Tianshan Mountain, respectively. Xinjiang belongs to a temperate continental arid and semi-arid climate, characterized by long hours of sunshine, strong evaporation, hot summers, and cold winters. In general, precipitation is higher in the north than in the south, and temperature is higher in the south than in the north. The vegetation cover is low, and the vast majority of the area is covered by desert, followed by grassland, which accounts for 70.90% and 22.95% of the total area, respectively.

### 2.2 Data

The remote sensing data used in this study include MODIS vegetation index product (MOD13Q1) and MODIS land cover product (MCD12Q1) from 2001 to 2020. MOD13Q1 is a 250m resolution 16-day composite MODIS vegetation index product that provides two primary vegetation indices: NDVI and enhanced vegetation index (EVI). The multi-temporal composite algorithm selects the best available pixel values during the 16-day period to produce the composite vegetation index values, with the criteria of low cloud, low view angle, and the highest NDVI/EVI value. In this study, the NDVI was extracted and then monthly composited. MCD12Q1 is a 500m resolution annual MODIS land cover product that provides land cover data under five different classification schemes. It is derived using supervised classification methods based on Terra and Aqua MODIS reflectance data and ancillary information. In this study, the land cover using the International Geosphere–Biosphere Programme (IGBP) scheme was used. In addition, the Shuttle Radar Topography Mission (SRTM) Digital Elevation Model (DEM) was also employed in this study to provide 90m resolution elevation.

The meteorological reanalysis data is the ERA5-Land meteorological data, which combines model data with observation data into a globally complete and consistent dataset using the laws of physics. It provides a series of meteorological elements with a spatial resolution of 0.1 degree. In this study, monthly mean temperature and monthly total precipitation from 2001 to 2020 were extracted.

The observed monthly sunlight hours and precipitation data were collected from 53 meteorological stations in Xinjiang. The sunlight hours were interpolated to gridded data with a spatial resolution of 1km based on the ordinary kriging method. Compared with the reanalysis precipitation and meteorological observed precipitation, the reanalysis precipitation is generally higher than observed precipitation. To reduce the error of reanalysis precipitation, observed precipitation from 53 meteorological stations in Xinjiang were employed to develop linear regressions between the reanalysis precipitation and meteorological observed precipitation month by month. Then the reanalysis precipitation were adjusted based on these equations.

All data were processed in Google Earth Engineering (GEE) platform, including mosaic, spatial clip, projection conversion, mask, monthly composite processing, spatial resampling, etc. Finally, all data were converted into Albers projection with a spatial resolution of 500m.

## 3 Methods

### 3.1 NPP estimation

The CASA (Carnegie–Ames–Stanford approach) model was employed for NPP estimation. It has the advantages of high accuracy and fewer input parameters and, and has been widely used for NPP estimation. Its main formula is:


(1) $$NPP_{\left(x,t\right)}=APAR_{\left(x,t\right)}\cdot \epsilon_{\left(x,t\right)}$$

where APAR_(x,t)_ is the absorbed photosynthetic active radiation of pixel x in month t (MJ·m^-2^); ϵ_(x,t)_ is light utilization efficiency of pixel x in month t (gC·MJ^-1^).

APAR is calculated by the following equation:


(2) $$APAR_{\left(x,t\right)}=SOL_{\left(x,t\right)}\cdot FPAR_{\left(x,t\right)}\cdot 0.5$$

Where SOL_(x,t)_ is the total solar radiation of pixel x in month t (MJ·m^2^), FPAR is the fraction of absorbed photosynthetically active radiation.

The total solar radiation was calculated based on the empirical equation proposed by He and Xie (2010), which was developed for Western China:


(3) $$Q=Q_{0}\left(a+b\cdot T_{S}/T_{A}\right)$$

Where Q_0_ is the astronomical radiation, which is calculated based on geographical latitude, solar declination and other parameters; T_S_ is the observed sunlight hours; T_A_ is the theoretical sunlight hours which is calculated from latitude and solar declination; a and b are empirical coefficients (a=0.185; b=0.595).

FPAR is calculated by Equation 4~7:


(4) $$FPAR_{\left(x,t\right)}=\alpha {\text{FPAR}}_{\text{NDVI}}+\left(1-\alpha \right){\text{FPAR}}_{\text{SR }}$$

(5) $${\text{FPAR}}_{\text{NDVI}}=\frac{{\text{NDVI}}_{\left(x,t\right)}-{\text{NDVI}}_{\left(i,min\right)}}{{\text{NDVI}}_{\left(i,max\right)}-{\text{NDVI}}_{\left(i,min\right)}}\times \left({\text{FPRA}}_{max}-{\text{FPRA}}_{\text{min }}\right)+{\text{FPRA}}_{\text{min }}$$

(6) $${\text{FPAR}}_{\text{SR}}=\frac{{\text{SR}}_{\left(x,t\right)}-{\text{SR}}_{\left(i,min\right)}}{{\text{SR}}_{\left(i,max\right)}-{\text{SR}}_{\left(i,min\right)}}\times \left({\text{FPRA}}_{max}-{\text{FPRA}}_{\text{min }}\right)+{\text{FPRA}}_{\text{min }}$$

(7) $${\text{SR}}_{\left(x,t\right)}=\frac{1+{\text{NDVI}}_{\left(x,t\right)}}{1-{\text{NDVI}}_{\left(x,t\right)}}$$

Where NDVI_(i,min)_ and NDVI_(i,max)_ are the minimum and maximum values of NDVI for vegetation type i, respectively; the values of FPAR_min_ and FPAR_max_ were 0.001 and 0.95, respectively; SR_(i,min)_ and SR_(i,min)_ are the 5% and 95% percentile of NDVI values for vegetation type i, respectively.

The light utilization energy ϵ is calculated by the following equation:


(8) $$\epsilon_{\left(x,t\right)}=T_{\epsilon 1(x,t)}\cdot T_{\epsilon 2(x,t)}\cdot W_{\epsilon (x,t)}\cdot \epsilon_{max}$$

Where T_ϵ1_ and T_ϵ2_ represent the stressing effects of low and high temperatures on light energy utilization, respectively; W_ϵ(x,t)_ is the water stress effect coefficient; ϵ_max_ is the maximum light utilization efficiency under ideal conditions(gC·MJ^-1^).

**Table 1** gives the values of NDVI_(i,min)_, NDVI_(i,max)_, NDVI_(i,min)_ and NDVI_(i,max)_ and ϵ_max_ of different vegetation types (Zhu et al., 2007).

**Table 1:** The values of NDVI_(i,min)_, NDVI_(i,max)_, NDVI_(i,min)_ and NDVI_(i,max)_ and ϵ_max_ of different vegetation types.

Vegetation Type	NDVI_max_	NDVI_min_	SR_max_	SR_min_	ϵ_max_
Evergreen needleleaved forest	0.647	0.023	4.67	1.05	0.389
Deciduous coniferous forest	0.738	0.023	6.63	1.05	0.485
Deciduous broadleaf forest	0.747	0.023	6.91	1.05	0.692
Mixed forest	0.702	0.023	5.84	1.05	0.475
Shrubland	0.636	0.023	4.49	1.05	0.429
Grassland	0.634	0.023	4.46	1.05	0.542
Cropland	0.634	0.023	4.46	1.05	0.542
Water	0.634	0.023	4.46	1.05	0.542
Impervious surface	0.634	0.023	4.46	1.05	0.542
Barren	0.634	0.023	4.46	1.05	0.542

### 3.2 NEP calculation

Net Ecosystem Productivity (NEP) is defined as the difference between NPP and soil heterotrophic respiration:


(9) $$NEP=NPP-R_{H}$$

where NEP is net ecosystem productivity of vegetation (gC·m^-2^); NPP is net primary productivity of vegetation (gC·m^-2^); RH is soil heterotrophic respiration (gC·m^-2^).

Soil heterotrophic respiration is calculated by the empirical equation developed by Pei et al. (2003).


(10) $$R_{H}=3.069\cdot \left(e^{0.0912\cdot T}+\ln\left(0.3145\cdot R+1\right)\right)$$

Where RH is soil heterotrophic respiration (gC·m^-2^); T is air temperature (°C); R is precipitation (mm).

### 3.3 Spatio-temporal variations

The Theil-Sen Median (Sen) method was employed to determine the trend of NEP over the study area. This method is a robust nonparametric trend method, which does not require the data to follow a certain distribution (Fensholt et al., 2012; Zhang Z. et al., 2021). It has been widely used in the trend analysis of long-time data series.

The Sen’s slope value (β) indicates the magnitude of the NEP trend. A positive β value suggests an upward trend and a negative β value suggests a downward trend. The calculation formula for β is as follows:


(11) $$\beta =\text{Median}\left(\frac{NEP_{j}-NEP_{i}}{j-i}\right)\;\;\;2001\leq i<j\leq 2020$$

where β is Sen’s slope value; NEP_i_ and NEP_j_ are NEP in year i and j respectively.

The Mann–Kendall test is used to assess the significance of NEP trends. The test statistics S value is calculated as:


(12) $$S=\sum_{i-1}^{n-1}\sum_{j=1+1}^{n}\left(\mathrm{sgn}NEP_{j}\_NEP_{i}\right)$$

$$\text{sgn}\left(NEP_{j-}NEP_{i}\right)=\{\begin{array}{c} \begin{array}{c} 1\;\;NEP_{j-}NEP_{i}>0\\ 0\;\;NEP_{j-}NEP_{i}=0\\ -1\;\;NEP_{j-}NEP_{i}<0 \end{array} \end{array}$$

The test statistic Z value was obtained by standardizing S:


(13) $$Z=\{\begin{array}{cc} \frac{S-1}{\sqrt{\mathrm{var}\left(S\right)}} & S>0\\ 0 & S=0\\ \frac{S+1}{\sqrt{\mathrm{var}\left(S\right)}} & S<0 \end{array}$$

Where the function var is calculated as:


(14) $$\mathrm{var}(S)=\frac{(n(n-1)(2n+5)-\sum_{i=1}^{m}t_{i}(t_{i}-1)(2t_{i}+5))}{18}$$

where n is the number of sequence samples; m is the number of affiliated groups in the data sequence; t_i_ is the number of input values inside the affiliated group i.

To validate the significance of the trend, the absolute z-score value |Z| is compared with the critical value Z_1-α/2_ at a given significance level α. if |Z| is higher than Z_1-α/2_, the trend is considered significant. The Z_1-α/2_ values were obtained from the standard normal distribution table. For the significance level α of 0.05 and 0.01, the critical Z_1-α/2_ values are 1.96 and 2.58, respectively

Partial correlation is used to analyze the relationship between NEP and meteorological factors. Its formula is:


(15) $${\text{r}}_{\text{ij}\cdot \text{k}}=\frac{{\text{r}}_{\text{ij}}-{\text{r}}_{\text{ik}}{\text{r}}_{\text{jk}}}{\sqrt{\left(1-{\text{r}}_{\text{ik}}^{2}\right)\left(1-{\text{r}}_{\text{jk}}^{2}\right)}}$$

where R_ij,k_ is the partial correlation coefficient between variable i and j after variable k is fixed. r_ij_, r_ih_, r_jh_ are correlation coefficients for the variables i and j, j and k, and k and i, respectively.

## 4 Results and discussions

### 4.1 Spatial distributions of NEP

**Figure 2:**
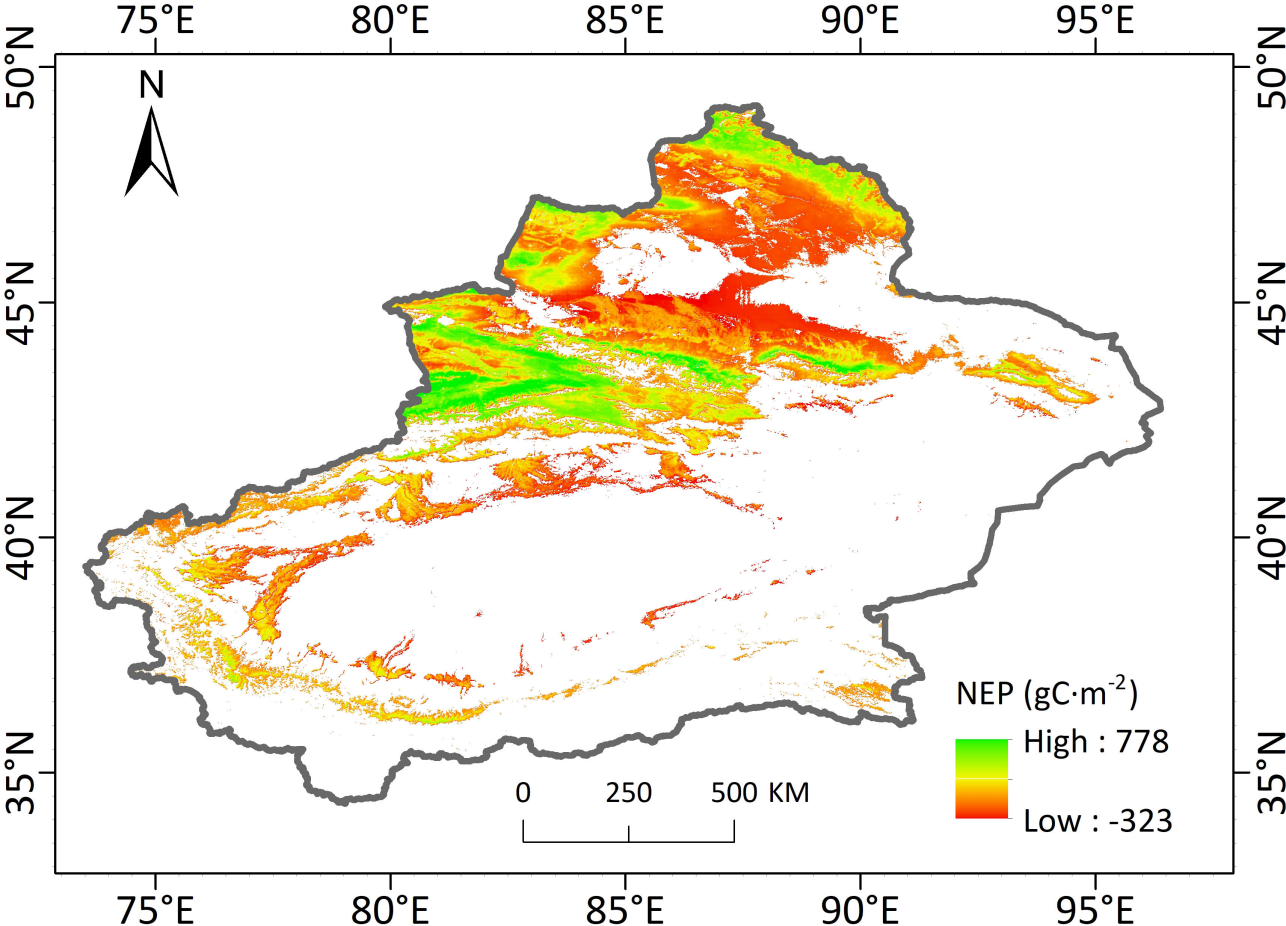
Spatial distribution of 20-year mean NEP in Xinjiang.

**Figure 2** shows the spatial distributions of the 20-year mean NEP of Xinjiang Autonomous Region. NEP in Xinjiang shows a spatial distribution pattern of high in the east and low in the west, high in the north and low in the south. During 2001-2020, the overall average vegetation NEP of the study area was 128.54 gC·m^-2^, suggesting an overall performance of carbon sink. However, the spatial differences were obvious. 61.56% of the vegetation area in the region had positive NEP, showing carbon sink effects. 38.44% of the vegetation area had negative NEP, showing a carbon source effect. The carbon source is mainly distributed in the central Junggar Basin, Altay Mountain, and southern Tacheng Basin.

### 4.2 Temporal variations of NEP

**Figure 3:**
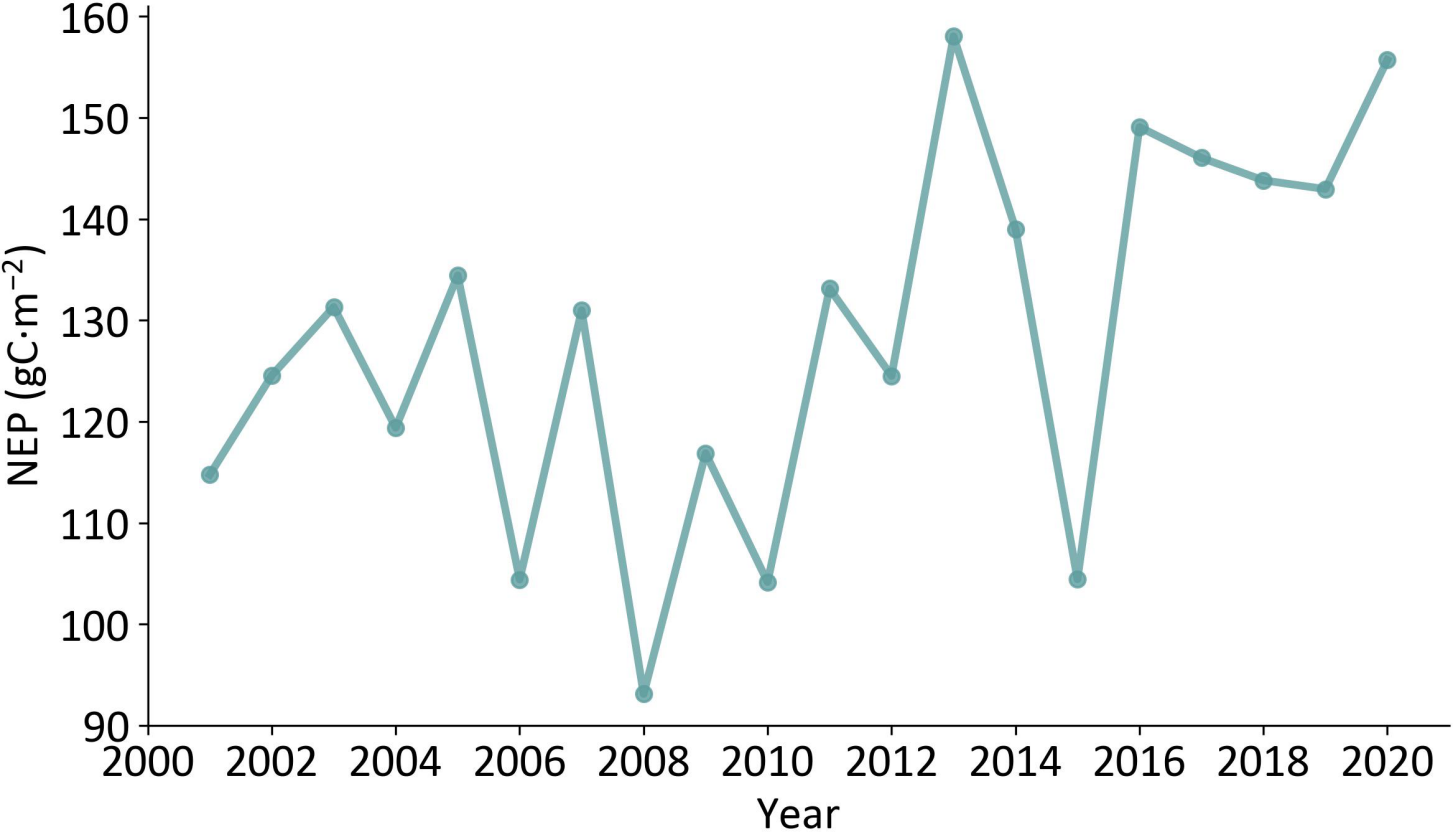
Temporal variations of annual mean vegetation NEP over Xinjiang from 2001 to 2020.

**Figure 3** shows the temporal variations of annual mean vegetation NEP over Xinjiang from 2001 to 2020. Generally, the annual mean vegetation NEP ranged from 93.12 to 158.05 gC·m^-2^, exhibiting an increasing trend with a Sen’s slope of 1.59 gC·m^-2^·a^-1^. During these 20 years, the annual mean vegetation NEP was the lowest in 2008 (93.12 gC·m^-2^) and the highest in 2013 (158.05 gC·m^-2^).

**Figure 4:**
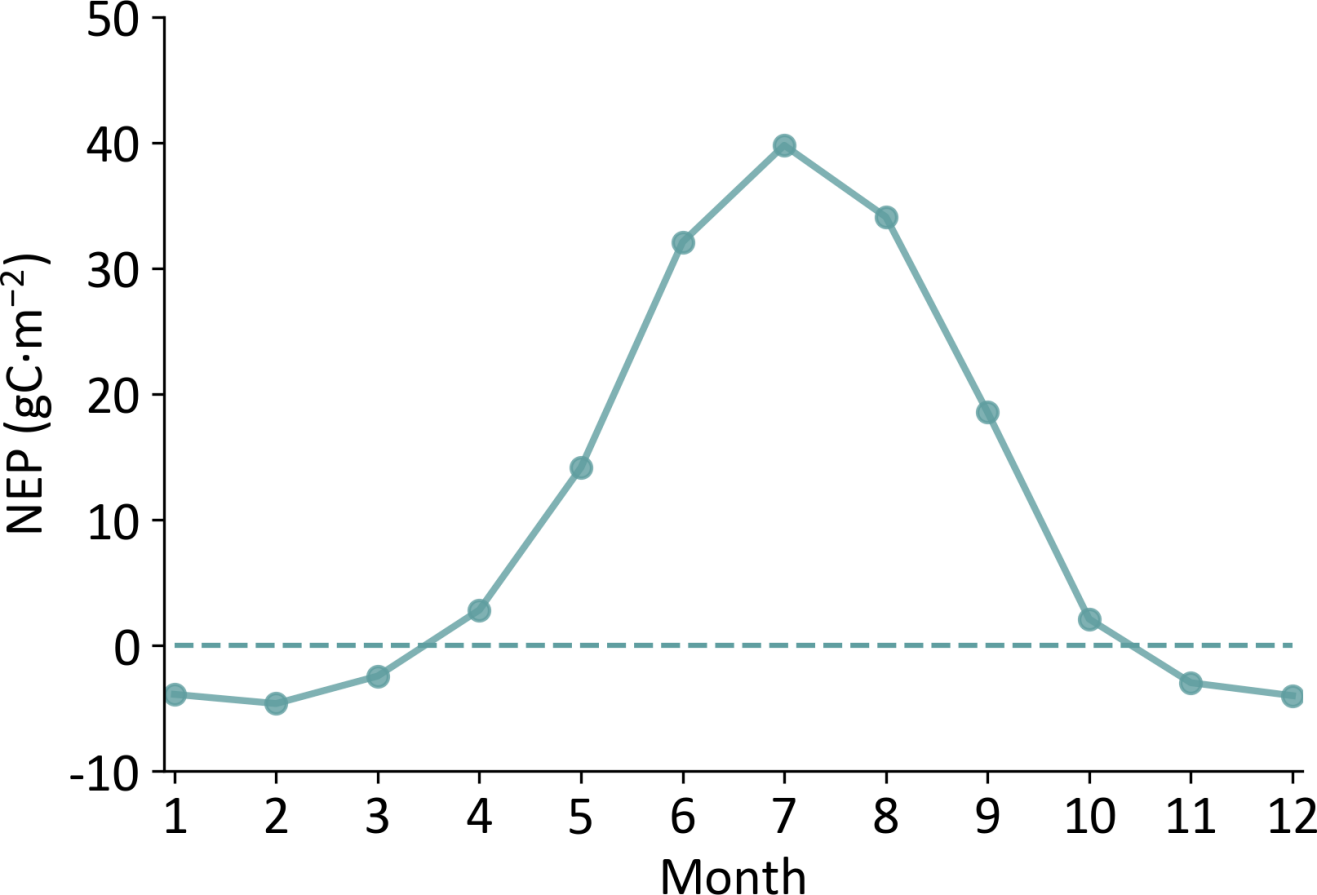
Temporal variations of monthly mean NEP over Xinjiang.

The monthly mean vegetation NPP in Xinjiang ranged from -4.62 to 39.81 gC·m^-2^, showing obvious single-peaked characteristics (**Figure 4**). Among the 12 months, NEP showed positive values from April to October and achieved the highest value in July (39.81 gC·m^-2^), indicating that during these months Xinjiang vegetation ecosystem acted as a carbon sink. From November to March, Xinjiang vegetation ecosystem showed a carbon source effect, and the lowest monthly NEP value was -4.62 gC·m^-2^ in February.

**Figure 5:**
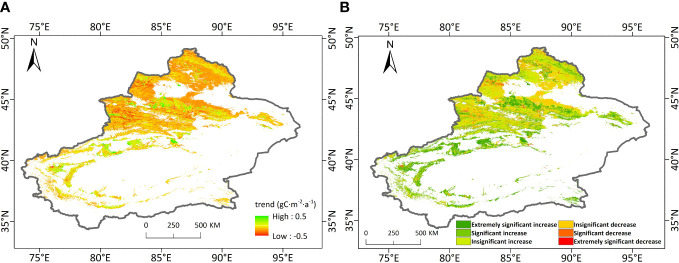
Trend of NEP **(A)** and the significance of the trend **(B)** over Xinjiang from 2001 to 2020.

To better understand the spatial and temporal variations of NEP over the study area, the Sen’s trend analysis and Mann–Kendall test were performed pixel by pixel. **Figure 5** gives the spatial distributions of the trend of vegetation NEP and the significance of the trend over Xinjiang from 2001 to 2020. During this period, most of the vegetation area in the region showed increasing trends. The area with increasing NEP trend was ~327, 400 km^2^, accounting for 71.46% of the total vegetation area. Among these areas, about 51.63% had the NEP trend lower than 0.15 gC·m^-2^·a^-1^, 21.65% had the NEP trend between 0.15 and 0.25 m^-2^·a^-1^, and 20.09% had the NEP trend between 0.25 and 0.5 gC·m^-2^·a^-1^. 6.64% had NEP growth rates greater than 0.5 gC·m^-2^·a^-1^, mainly in the oasis belt of the northern slopes of the Tianshan Mountain, the oasis belt around the Tarim Basin, the Altai Mountain, and the northern part of the Tacheng Basin. The area where NEP showed decreasing trends was ~130, 800 km^2^, accounting for 28.54% of the total vegetation area. Most of these areas had NEP decline rates below 0.15 gC·m^-2^·a^-1^, accounting for about 79.61% of the vegetation area with decreasing NEP trends. These areas were mainly located in the Ili Basin, the northern part of the Junggar Basin, the Altai Mountain and the south part of the Tacheng Basin. 11.34% had NEP decline rates between 0.15 and 0.25 gC·m^-2^·a^-1^, 7.92% had NEP decline rates between 0.25 and 0.5 gC·m^-2^·a^-1^. Only 1.13% had NEP decline rates over 0.5 gC·m^-2^·a^-1^, which were sporadically distributed throughout the region. In terms of the distribution of significant levels of trends (**Figure 5B**), 22.33% of the vegetation area in Xinjiang showed highly significant increasing trends, mainly in the oasis belt in the middle of the northern slope of the Tianshan Mountain and the oasis belt around the Tarim Basin. There were also 11.01% of vegetation area showed significant increasing trends. Most of the vegetation area in the study area showed insignificant changes, accounting for 63.23% of the total vegetation area. Among them, 38.12% had insignificantly increasing trends and 25.11% had insignificantly decreasing trends. Only 2.16% and 1.27% of the vegetation area showed significant decreasing and highly significant decreasing trends, respectively. In general, the area with increasing NEP was obviously larger than that with decreasing NEP, and the NEP decreasing trends were mostly insignificant, suggesting good vegetation recovery in Xinjiang.

### 4.3 Response of NEP to meteorological factors

**Figure 6:**
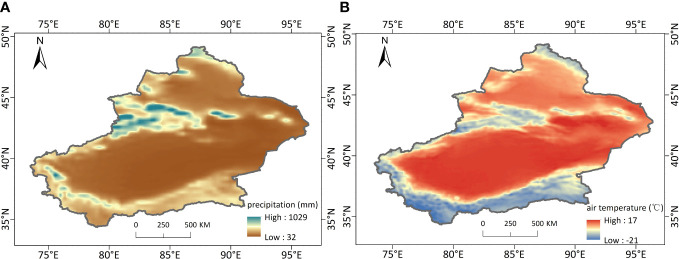
Spatial distributions of 20-year mean annual precipitation **(A)** and air temperature **(B)** over Xinjiang from 2001 to 2020.

**Figure 6** shows the 20-year average annual precipitation and temperature over Xinjiang. This regions exhibited strong spatial difference in precipitation. The Yili Basin region and the northern slopes of the Tianshan Mountain had the highest precipitation. This can be attributed to the fact that the Tianshan Mountain block the warm and humid Atlantic air currents, creating more precipitation on the windward slopes. And the open valley floor in the western part of the Yili Basin allows the entry of the humid Atlantic air currents. The Tarim Basin, Turpan Basin and Junggar Basin are surrounded by high mountains, making it difficult for water vapor to get in, resulting in low precipitation and the arid climate. The air temperature also varies widely among different regions, which is mainly influenced by the topography. The Altai Mountain, Tianshan Mountain, Kunlun Mountain and Ali Mountain have high altitude and low temperature, while the Junggar Basin, Ili Basin, Tarim Basin, Tacheng Basin and Turpan Basin have low altitude and high temperature. Compared with the spatial distribution of NEP, precipitation and temperature, the areas with high NEP overlapped with areas with high precipitation and low temperature to a high degree.

**Figure 7:**
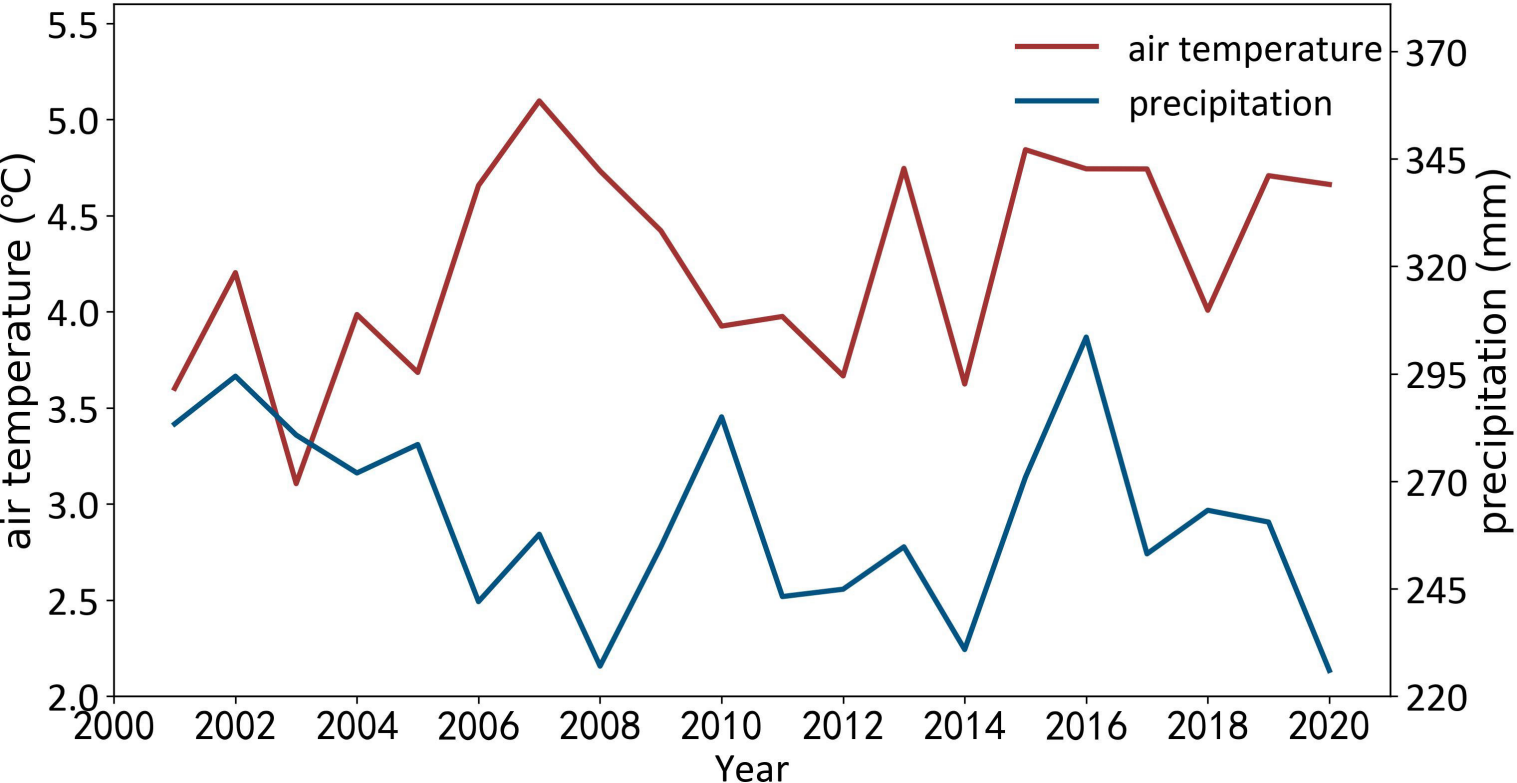
Temporal variations of annual precipitation **(A)** and air temperature **(B)** over Xinjiang from 2001 to 2020.

**Figure 7** shows the temporal variations of annual precipitation and air temperature over Xinjiang from 2001 to 2020. The annual precipitation of vegetation region ranged between 225.99 mm in 2020 and 303.53 mm in 2016, and the 20-year average annual precipitation was 261.32 mm. During this period, the annual precipitation showed an overall fluctuating decreasing trend. The annual temperature of vegetation region ranged from 3.11°C (2003) to 5.10°C (2007), and the 20-year average annual temperature was 4.26°C. Different from precipitation, the annual temperature exhibited an overall fluctuating increasing trend.

**Figure 8:**
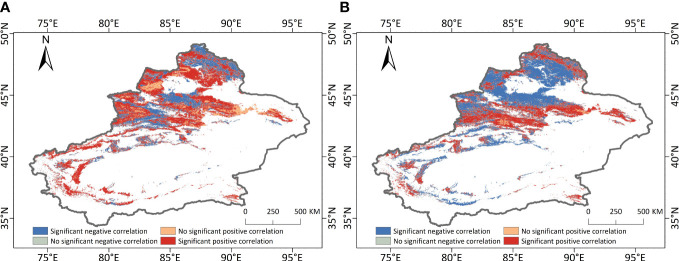
Spatial distributions of the significance of the partial correlation between annual NEP and precipitation **(A)** and air temperature **(B)** over Xinjiang from 2001 to 2020.

The overall partial correlation coefficient between vegetation NEP and precipitation in Xinjiang was 0.145, which showed a positive correlation in general, indicating that precipitation mainly contributed to vegetation NEP in the study area. The partial correlation analysis between NEP and climate factors (precipitation and air temperature) were also carried out pixel by pixel. **Figure 8** gives the spatial distribution of the significance of the partial correlation between annual NEP and the two climate factors. 52.57% of the vegetation NEP showed significant positive correlations with precipitation, and 15.66% of the vegetation NEP showed insignificant positive correlations with precipitation. The areas with negative correlation between vegetation NEP and precipitation are relatively small, of which 29.74% show significant negative correlations and 2.04% show insignificant negative correlations, mainly in the oasis zones around the Tarim Basin, the Tianshan Mountains in the north and south of the Ili Valley, the Altay Mountains and the southern side of the Junggar Basin. Precipitation in these regions showed decreasing trends over the past 20 years, while NEP showed increasing trends. This can be attributed to the implementation of ecological water replenishment, afforestation and other ecological protection projects in Xinjiang Autonomous Region, which resulted in abundant water resources and less influence by natural precipitation. Moreover, the decrease in precipitation reduced soil microbial respiration, therefore increased organic carbon consumption and therefore increased vegetation NEP. The partial correlation coefficient between vegetation NEP and annual air temperature in Xinjiang was -0.0368, suggesting that high temperature leads to low NEP. Most of the vegetation NEP showed significant negative correlation with temperature, accounting for 52.77% of the total vegetation area in the study area. The increase in temperature increased the transpiration of vegetation, intensified the evaporation rate of soil water, and reduced the NPP accumulated by vegetation, while the increase in temperature also increased the respiration of soil microorganisms, therefore the vegetation NEP value decreased. 40.84% of the vegetation NEP was significantly and positively correlated with temperature, mainly in the Tianshan Mountains and Altai Mountains. This can be attribute to the fact that higher temperature produces more alpine snow and ice melt water, which increases the amount of water in the region, promotes the growth and development of vegetation and enhances the vegetation’s carbon sequestration capacity. Only 2.57% of the vegetation NEP showed insignificant positive correlation and 3.82% showed significant negative correlation with temperature. Generally, both precipitation and temperature had an influence on NEP in Xinjiang, and temperature had less influence than precipitation.

## 5 Discussions

Though a lot of studies have been carried out on mapping NPP and studying the spatial and temporal variations, the studies on the spatiotemporal variations of NEP are much fewer. Compared with NPP, NEP can better indicate the carbon absorption and emission capacity, which are more meaningful for carbon cycle research. This study is the first research that estimates NEP in Xinjiang, a typical ecologically fragile area in China, providing valuable knowledge for understanding the regional carbon sequestration capacity and for developing strategies to stabilize CO_2_ emissions.

Previous studies generally use station-based observations to produce spatial continuous meteorological factors to estimate NEP. However, in Xinjiang there are quite a few meteorological stations and the land and atmospheric characteristics have obvious spatial differences. Spatial interpretation cannot well depict the spatial variations of meteorological factors. Based on this consideration, ERA5-land reanalysis data were employed in this study to derive gridded precipitation and air temperature, which can provide much more spatial details than interpolated meteorological observations in the study area. This study also has a limitation that there is no *in-situ* NPP observation data for validation. We compared our results with that of the studies of adjacent regions, such as Qinghai Province and Inner Mongolia Autonomous Region. The NEP values for typical vegetation types are similar. Moreover, the CASA model has been widely used in many regions and has proven its applicability. Therefore, the estimated NEP results are credible for the spatial and temporal variations.

Despite the relatively small vegetation coverage in Xinjiang, the terrestrial ecosystem still exhibits an overall carbon sink effect from 2001 to 2020. Moreover, the terrestrial carbon sink showed an obvious increasing trend in the past 20 years. The partial correlation analysis between annual NEP, precipitation, and air temperature indicated that NEP was positively correlated with precipitation and negatively correlated with temperature. Precipitation exerted a stronger influence than temperature, indicating that precipitation is the dominant driving factor that influences the temporal trend of carbon sequestration capacity in this arid ecosystem.

## 6 Conclusions

By integrating satellite remote sensing data and reanalysis meteorological data, the spatio-temporal variations of NEP in Xinjiang Autonomous Region, China from 2001 to 2020 were studied based on the CASA model and the soil heterotrophic respiration model. The NEP in Xinjiang showed a spatial pattern that was high in the east and low in the west, high in the north and low in the south. The overall annual mean vegetation NEP of 128.54 gC·m^-2^. During the past 20 years, the annual mean NEP over Xinjiang increased with the Sen’s slope of 1.59 gC·m^-2^·a^-1^, indicating that the carbon sink effect of the vegetation ecosystem in Xinjiang was enhanced. The trend of NEP also exhibited a large spatial heterogeneity. The area with increasing NEP trend accounted for 71.46% of the total vegetation area. Annual NEP was positively correlated with precipitation but negatively correlated with temperature. Compared with temperature, NEP was more sensitive to precipitation in this region. This study provides a reference for the sustainable development of the terrestrial ecosystem and the impact of climate change on the carbon cycle in Xinjiang, and also provides a template for NEP investigation in other regions.

## Data availability statement

The raw data supporting the conclusions of this article will be made available by the authors, without undue reservation.

## Author contributions

XL carried out the study and wrote the manuscript; YC and YS collected and processed the data, provided suggestions and edited the manuscript; YoX designed the work, helped organize the manuscript structure, and directed the study; YaX drew the figures; YM provided suggestions. All authors contributed to the article and approved the submitted version.
